# PepAnno: A structure-aware deep learning framework for bioactive peptide prediction, structural visualization, and physicochemical profiling

**DOI:** 10.1371/journal.pcbi.1014369

**Published:** 2026-06-02

**Authors:** Enyan Liu, Yueming Hu, Liya Liu, Yifan Chen, Shilong Zhang, Sida Li, Haoyu Chao, Luyao Xie, Yi Shen, Liangwei Wu, Cong Feng, Julio Raúl Fernández Massó, Ming Chen

**Affiliations:** 1 Department of Bioinformatics, College of Life Sciences, Zhejiang University, Hangzhou, China; 2 Anhui Province Key Laboratory of Embryo Development and Reproductive Regulation, School of Biological and Food Engineering, Fuyang Normal University, Fuyang, China; 3 Institute of Hematology, The First Affiliated Hospital, Zhejiang University School of Medicine, Hangzhou, China; 4 Pharmaceutical Department, Center for Genetic Engineering and Biotechnology, Havana, Cuba; 5 State Key Laboratory of Vegetation Structure, Function and Construction, Zhejiang University, Hangzhou, China; 6 Zhejiang Key Laboratory of Multi-omics Precision Diagnosis and Treatment of Liver Diseases, Zhejiang University, Hangzhou, China; Fudan University, CHINA

## Abstract

Peptides are gaining prominence as therapeutic candidates due to their diverse physiological functions and structural simplicity. Although multiple computational tools exist for bioactive peptide prediction, many suffer from limitations such as non-intuitive interfaces, sequence-only representations, insufficient structural awareness, restricted interpretability, or fragmented analysis workflows, leading to reduced research efficiency and higher costs. To address these challenges, we present PepAnno (https://bis.zju.edu.cn/pepanno/), a comprehensive and user-friendly web server for multi-functional peptide annotation. PepAnno is powered by a novel structure-aware, multi-view geometric deep learning framework that integrates pre-trained sequence embeddings with predicted 3D structural graphs through a dual-stream architecture combining a Transformer and a GATv2 network. A cross-modal attention mechanism is employed to effectively fuse semantic and geometric representations, enabling accurate multi-task prediction across 7 key bioactivities, including antimicrobial and anticancer properties. Comprehensive evaluation on seven curated bioactivity datasets demonstrates that PepAnno achieves robust and competitive predictive performance across tasks, consistently outperforming or matching existing methods in terms of discrimination and stability. Beyond functional prediction, PepAnno provides automated calculation of physicochemical properties, structure visualization, and access to an integrated repository of peptide-related databases and tools. By enabling one-click peptide annotation, PepAnno offers an efficient and interpretable solution for large-scale peptide analysis and facilitates downstream experimental design and peptide-based drug discovery.

## Author summary

PepAnno is an integrated web server developed to advance the study of bioactive peptides—small yet versatile molecules with significant therapeutic and diagnostic potential. Although several computational tools have been developed to identify peptide activities, researchers often need to rely on multiple independent platforms to obtain functional, structural, and physicochemical information, resulting in fragmented and inefficient workflows. More importantly, most existing predictors operate as black boxes, offering limited mechanistic insight into how specific spatial motifs govern biological functions. To bridge this gap, we developed PepAnno, a comprehensive and user-friendly web server. PepAnno is powered by a novel structure-aware, multi-view deep learning framework that synergizes sequence semantics with 3D structural geometry. By leveraging a strict hierarchical transfer learning strategy, it achieves highly accurate predictions across seven major functional categories, effectively overcoming the challenge of data scarcity. Crucially, PepAnno breaks the barrier by providing native biological interpretability. It dynamically maps the model’s cross-attention weights onto 3D structures, empowering researchers to visually pinpoint key functional residues. Along with automated physicochemical profiling and a curated knowledge base of peptide resources, PepAnno unifies robust prediction, structural interpretability, and centralized data access. This integrated design significantly streamlines research workflows, helping scientists formulate mechanistically meaningful hypotheses and accelerating peptide-based drug discovery.

## Introduction

Bioactive peptides (BPs) are short-chain molecules formed by amino acids linked via peptide bonds, widely distributed in various biological organisms, including animals and plants [1]. BPs exhibit a diverse array of biological activities, encompassing crucial functions such as antimicrobial, anticancer, anti-inflammatory, and antiviral effects [2–5]. For instance, antimicrobial peptides (AMPs), a class of short peptides with broad-spectrum antimicrobial, antiviral, and antifungal activities, are ubiquitously found in the epithelial barriers and systemic immune defense systems of multicellular eukaryotes [3,6]. Compared to conventional single-target antibiotics, AMPs possess a relatively lower risk of inducing microbial resistance, attributable to their rapid and efficient membrane-acting mechanisms and multi-target inhibitory properties [6]. Beyond AMPs, other BPs also hold substantial clinical promise, driving extensive research into their classification and functional characterization [7,8]. Over the past few decades, more than 7,000 naturally occurring peptides with extensive biological activities have been identified within the human body. These peptides typically exert their biological effects by binding to cell surface receptors (particularly G protein-coupled receptors), thereby activating intracellular signal transduction pathways [9]. Their short sequence lengths (typically <50 residues) further facilitate chemical synthesis, making BPs ideal candidates for novel therapeutics and diagnostics [10,11]. The rapid advancements in molecular biology and bioinformatics have further underscored the therapeutic potential of peptides, establishing BPs as a key research focus in contemporary life sciences and medicine [12]. Nevertheless, owing to their high sequence diversity, the accurate identification and functional prediction of BPs remain significant challenges, particularly in high-throughput screening processes where associated costs can also be considerable.

The rapid accumulation of experimental data in peptide omics and related fields has stimulated the development of machine learning approaches for bioactive peptide (BP) function prediction, resulting in a growing number of computational tools [13–15]. In particular, the identification of multifunctional BPs is inherently a multi-label classification problem, motivating the adoption of multi-label learning strategies [16–18]. Despite these advances, the application of multi-label and multi-functional models to BP prediction remains constrained. Existing approaches often exhibit reduced predictive accuracy as the number of functional categories increases, largely due to their reliance on sequence-only representations and extensive zero-padding of variable-length peptides. Such strategies may obscure biologically meaningful signals and limit the modeling of function-specific structural determinants. Moreover, most current multi-functional platforms provide limited interpretability and lack structure-aware or residue-level insights that are critical for understanding peptide function mechanisms. From a practical perspective, both single-function and multi-functional BP prediction tools continue to face challenges in usability and sustainability. Our survey of 135 BP prediction tools published within the past five years revealed that many suffer from fragmented workflows, incomplete documentation, unavailable or non-callable source code, and discontinued online services. Even when local deployment is feasible, users often need to combine multiple independent tools to obtain complementary functional and structural information, making comprehensive peptide analysis inefficient and error-prone.

To overcome the aforementioned limitations, we developed PepAnno, a structure-aware, multi-functional peptide annotation platform that unifies sequence analysis, structural modeling, and functional prediction within a single framework. PepAnno enables “one-click” automated analysis, ranging from physicochemical property calculation and structure prediction to the annotation of seven major bioactive peptide functions, including antimicrobial, anticancer, anti-inflammatory, antiviral, antihypertensive, anti-angiogenic, and cell-penetrating activities. By integrating structure-aware learning and cross-modal feature fusion, PepAnno provides accurate and interpretable predictions while substantially simplifying peptide analysis workflows. In addition, PepAnno incorporates a curated repository of manually validated peptide-related databases and computational resources, offering a centralized and freely accessible platform to support systematic peptide research and downstream applications.

## Results

### Functionality of PepAnno

PepAnno serves as integrated web-based platform for peptide sequence annotation and functional analysis (Fig 1). Its primary functionality is the AI-driven evaluation of peptide bioactivities. Furthermore, the platform facilitates the calculation of fundamental physicochemical properties of peptides and enables the prediction and visualization of secondary and tertiary structures.

**Fig 1:**
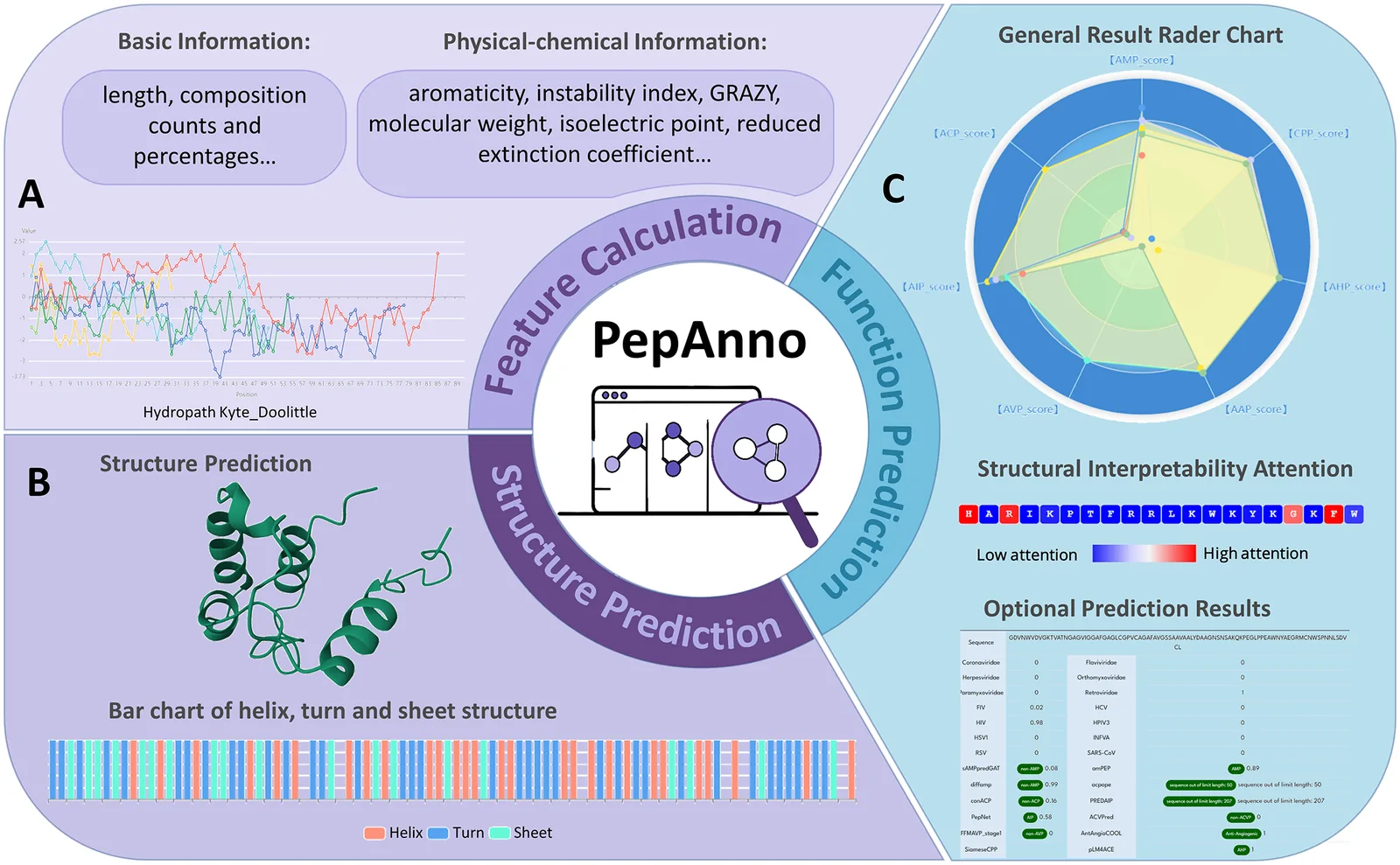
Overview of the PepAnno platform’s functionalities. The platform is organized into three main modules: **(A) Feature Calculation:** Peptide feature calculation, encompassing basic information and physicochemical properties. **(B) Structure Prediction:** Structure prediction, which includes calculating scores for secondary structure elements and predicting tertiary structures. **(C) Function Prediction:** Bioactive function prediction, covering seven key activities with structural interpretability attention.

Users initiate predictions via the ‘Predict’ interface or the ‘Other Tools’ interface (see Fig A in S1 Appendix). In the prediction interface, Users can select from 54 amino acid scales for profile computation (e.g., Hydropathicity Scale, Transmembrane tendency scale by default) and adjust the compute window size. For optional model-based predictions, users can select from seven types of peptide bioactive functions and choose specific predictive models.

PepAnno provides comprehensive visualization of prediction results organized into four modules (see Fig A in S1 Appendix). **‘General Information’** provides a summary table of basic peptide attributes, accompanied by visualizations such as amino acid composition bar charts and residue percentage line plots. **‘Physical-chemical Information’** presents key physicochemical properties (e.g., molecular weight, aromaticity, instability index and isoelectric point) in a structured tabular format with line charts illustrate trends based on user-selected protein scale. **‘Structural Information’** summarizes predicted secondary structure content (α-helix, β-turn, and β-sheet) in tabular form and visualizes residue-level secondary structure propensities using bar charts. In addition, PepAnno generates interactive three-dimensional structure models with downloadable PDB files for tertiary structure analysis. **‘Bioactive Function’** provides a general results table and a radar chart displaying predicted activities for all input peptides. For each peptide, detailed prediction scores are presented, along with the interpretable sequence attention. For predictions generated using optional models, an additional integrated table summarizes scores across the selected methods.

In response to the rapid expansion of peptide-related databases and computational design tools, PepAnno also provides a ‘Resources’ module that integrates curated peptide research resources, including databases, web analysis platforms, and computational tools. This module presents a table detailing each resource’s name, key features, description, access link, and associated publication link. Users can filter resources by type (Database, Webserver, or Tool), and a top 10 feature frequency summary highlights commonly represented functionalities to aid efficient resource discovery. In addition, datasets used by PepAnno and other collected peptide datasets are made available for download through the ‘Data’ interface.

### Ablation studies

To explicitly isolate the incremental value of our training strategies and architectural components, we conducted a targeted ablation study on the representative AVP task. All variants were evaluated under a rigorous 5-fold cross-validation protocol, reporting the mean and standard deviation to ensure stability comparisons (Table 1).

**Table 1:** Ablation Study on the AVP Task (5-Fold Cross-Validation).

Model Variant	Architecture	Training Strategy	ACC	SN	SP	MCC	AUC
Full Model	Seq + Struct GNN	Pretrain + Head Reset	0.8663 ± 0.0096	0.8773 ± 0.0161	0.8555 ± 0.0284	0.7335 ± 0.0186	0.9249 ± 0.0095
Variant A	Sequence Only	Direct Training	0.8742 ± 0.0075	0.8716 ± 0.0333	0.8767 ± 0.0223	0.7494 ± 0.0145	0.9430 ± 0.0053
Variant B	Seq + Struct GNN	Direct Training	0.8581 ± 0.0028	0.8445 ± 0.0112	0.8715 ± 0.0117	0.7165 ± 0.0055	0.9202 ± 0.0080
Variant C	Seq + Struct GNN	Pretrain + No Reset	0.8650 ± 0.0082	0.8651 ± 0.0183	0.8650 ± 0.0096	0.7303 ± 0.0165	0.9274 ± 0.0068

Comparing the full model with Variant B (Direct Training), we observed a significant performance degradation in Variant B. This validates that pre-training on the large-scale AMP dataset is indispensable for establishing a robust feature backbone and preventing overfitting on smaller datasets. Furthermore, evaluating Variant C (Pretrain + No Reset) highlights the necessity of the Head Reset strategy. Although keeping the pre-trained classification head (Variant C) yielded a comparable AUC, resetting the head (Full Model) resulted in a superior and more stable MCC (0.7335 vs. 0.7303) and Accuracy (0.8663 vs. 0.8650). This confirms that resetting task-specific decision boundaries effectively mitigates negative transfer between orthogonal peptide functions. We also evaluated a sequence-only model (Variant A). Driven by the massive representational capacity of the pre-trained ProtT5 language model, this simplified variant achieved slightly higher scores on certain metrics. However, it exhibited a lower true positive rate (Sensitivity) compared to our Full Model (0.8716 vs. 0.8773). Furthermore, sequence-only predictions lack the ability to anchor its predictions in spatial physical geometry.

### Performance

#### Holistic performance evaluation.

A holistic evaluation of the model’s classification capability is presented in Fig 3A, complemented by the comprehensive performance summary in Tables B in S1 Appendix. As illustrated in Fig 3A, the model demonstrates a well-balanced performance profile across multiple evaluation metrics, including AUC, Accuracy, and F1-score. Notably, the model achieves exceptional discriminative power on AVP and CPP tasks, with AUC values exceeding 0.90, highlighting its strong capability to distinguish antiviral and cell-penetrating peptides from non-functional sequences. For more challenging categories such as AAP and AIP, where limited sample availability and higher functional heterogeneity typically impede deep learning performance, the model maintains competitive accuracy and F1-score values without excessive degradation.

To rigorously verify that our framework generalizes robustly and is not biased toward specific sequence lengths, we conducted a length-stratified evaluation on the independent test set (using the AVP task as a representative case). The test sequences were partitioned into three subgroups based on length(L): Short (L ≤ 10), Medium (11 ≤ L ≤ 25), and Long (L > 25). As illustrated in Fig 2, PepAnno maintained highly consistent predictive performance (AUC and ACC) across all length strata.

**Fig 2:**
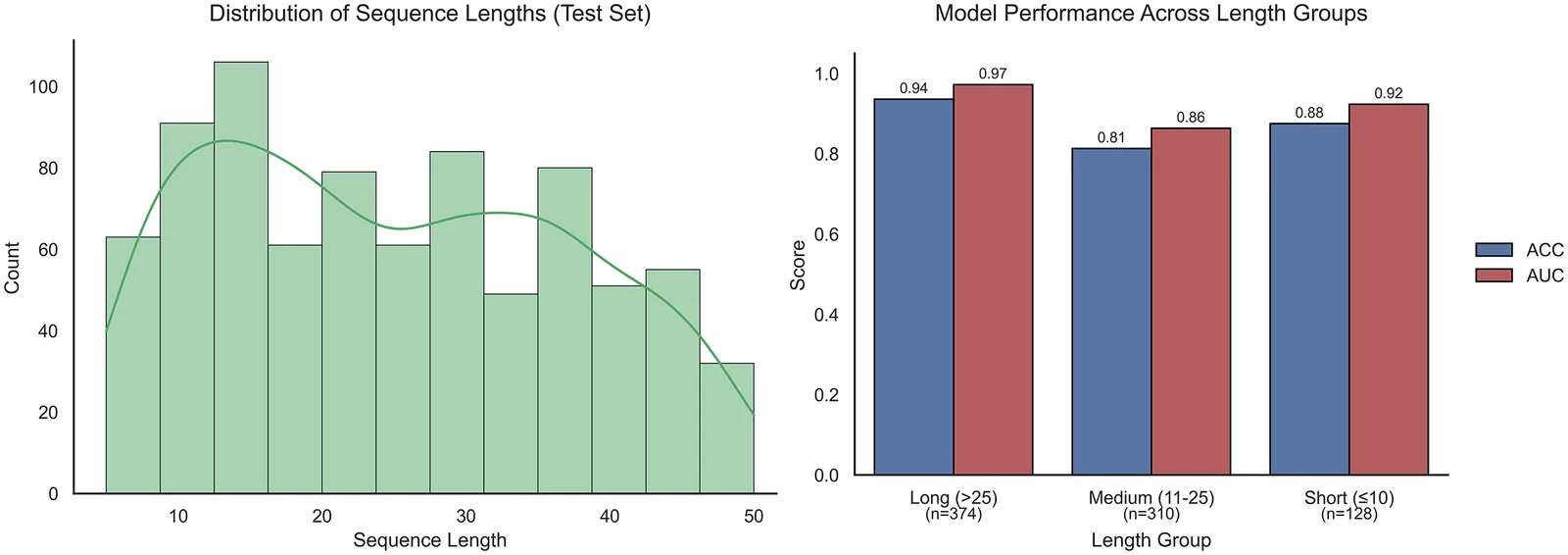
Length-stratified evaluation on the AVP independent test set.

**Fig 3:**
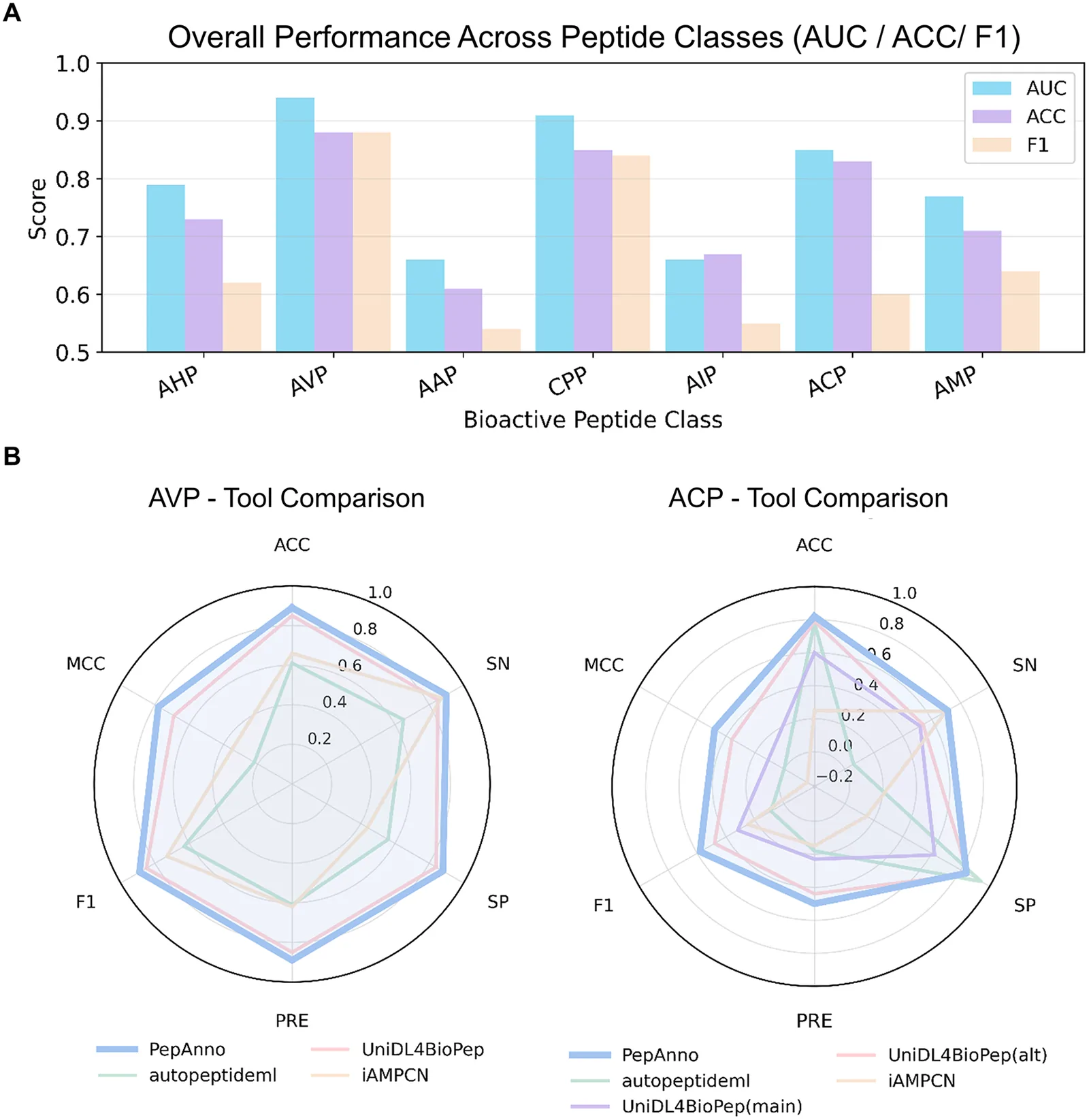
Model performance. **(A)** Overall performance (AUC, ACC, and F1-score) of the proposed model across seven peptide categories on the independent test dataset. **(B)** Radar chart comparisons of PepAnno and existing tools on the AVP and ACP categories. PepAnno is highlighted for clarity.

Detailed training dynamics and convergence behavior across folds are provided in S1 Appendix (Fig B, C and Table A), further supporting the robustness and stability of the proposed framework. Detailed definitions of the model evaluation metrics are given in S1 Appendix.

#### Comparison with state-of-the-art methods.

To comprehensively evaluate PepAnno, we conducted two complementary benchmarking analyses: (1) comparisons with task-specific predictors within each of the seven functional categories, and (2) comparisons with existing multi-functional peptide prediction platforms. For category-wise evaluations, PepAnno was benchmarked against representative state-of-the-art tools specifically designed for each activity, enabling a fair assessment under matched task definitions and evaluation metrics. Across these comparisons, PepAnno consistently achieved competitive or superior performance, while operating under a unified multi-task framework rather than task-dependent feature engineering or model selection.

For multi-functional platform benchmarking, direct one-to-one comparison across all seven functional categories was not feasible, as no existing integrative predictor supports the identical functional spectrum covered by PepAnno. Therefore, we adopted an intersection-based evaluation strategy, restricting comparisons to functional categories shared between PepAnno and each multi-functional baseline. Specifically, PepAnno was compared with AutoPeptideML [13], iAMPCN [14], and UniDL4BioPep [15] on four overlapping functions, ensuring methodological consistency and avoiding extrapolation beyond the scope of each platform (Fig 3B, Tables C in S1 Appendix). Under this conservative setting, PepAnno demonstrated strong and balanced performance across shared tasks, matching or exceeding the predictive accuracy of existing multi-functional approaches while providing a broader functional coverage and residue-level interpretability not available in previous platforms.

Across seven category-specific benchmarks, PepAnno demonstrated consistently competitive performance relative to state-of-the-art task-specific predictors [14, 19–60]. It achieved top or near-top performance in antimicrobial (AMP), antiviral (AVP), antibiofilm-associated (AAP), and cell-penetrating peptide (CPP) prediction. For anticancer peptides (ACP), PepAnno ranked within the top tier, closely approaching leading specialized models. In more challenging categories with greater label heterogeneity, including anti-inflammatory (AIP), and antihypertensive peptides (AHP), PepAnno maintained competitive mid-range performance. Overall, these results indicate that a unified multi-task framework can effectively match specialized predictors across diverse peptide functions. Detailed results are provided in Tables D-J in S1 Appendix.

### Case study: Mechanistically interpretable multi-functional annotation of Human Neutrophil Peptide-1 (HNP-1)

Neutrophils are typically the first immune cells recruited to an infection site, where they release effector molecules such as Human Neutrophil Peptides (HNPs) [61]. Although HNPs exhibit direct and potent antimicrobial activities [62], these also modulate immune responses, including chemotaxis, phagocytosis, and cytokine induction. In addition to their antimicrobial functions, HNPs possess anticancer activities, including membranolytic and antiangiogenic effects [63].

We input the sequences of HNP-1 into PepAnno to perform a comprehensive analysis. The functional prediction module successfully validated the known antimicrobial and anticancer functions and, importantly, suggested novel potential activities, including anti-inflammatory, antivirus, anti-angiogenic and cell-penetrating activities, while assigning a negligible probability to antihypertensive activity (Fig 4A). The predicted antimicrobial and cell-penetrating activities are consistent with their primary mechanism, which involves electrostatic interactions between the cationic properties and anionic bacterial membrane, leading to membrane disruption. Similarly, the predicted anticancer function is supported by multiple lines of evidence, including the induction of membrane pores formation at high concentrations, inhibition of DNA synthesis, and interference with tumor angiogenesis. Crucially, the prediction of anti-inflammatory potential is particularly compelling, given that HNPs are known to modulate immune responses by regulating the release of inflammatory factors such as IL-8 [61,63].

**Fig 4:**
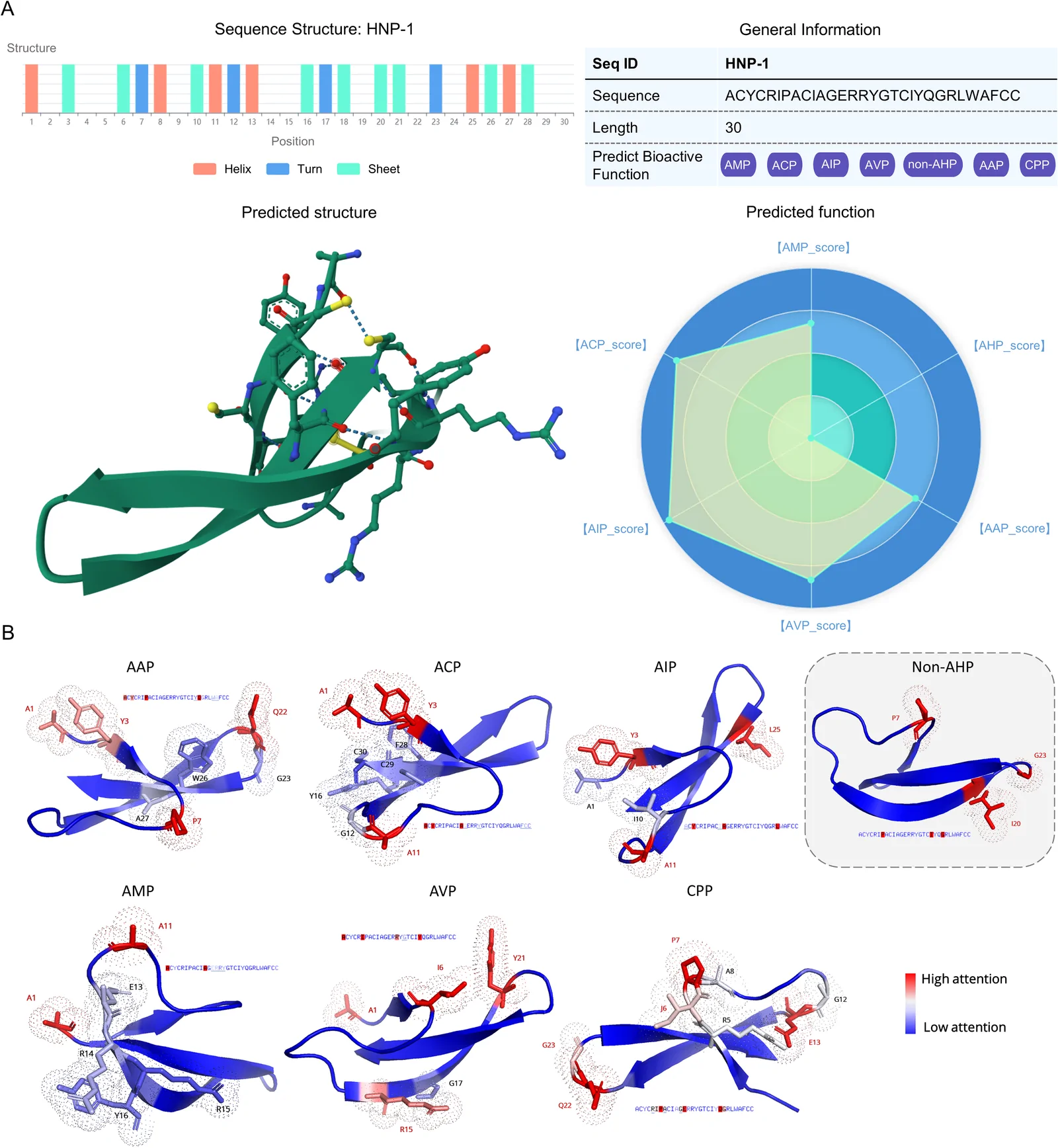
Case study of HNP-1. **(A)** Comprehensive multi-functional prediction of HNP-1 by PepAnno. **(B)** Residue-level attention patterns of HNP-1 across seven functional prediction heads.

Beyond function-level predictions, PepAnno enables residue-level interpretability by projecting attention weights from each functional prediction head onto the three-dimensional structure of HNP-1. Attention weights reflect the relative contribution of residues to model inference. As shown in Fig 4B, distinct functional heads emphasize partially overlapping yet clearly differentiated residue sets, revealing how the same peptide sequence can encode multiple biological activities through structurally localized determinants [64]. Among the seven evaluated bioactivities, antihypertensive (AHP) activity was the only function predicted as negative for HNP-1. In the results, residues A1 and A11 consistently receive high attention across several functions, reflecting their critical role in defining α-defensin identity and maintaining the correct β-sheet fold stabilized by conserved disulfide bonds. In contrast, antimicrobial and antiviral predictions preferentially highlight clusters of positively charged residues (e.g., R14 and R15), consistent with electrostatic interactions with anionic microbial membranes and viral envelopes. Importantly, functions with more specific mechanistic requirements display correspondingly distinct attention patterns. The antibiofilm-associated and anticancer predictions strongly emphasize hydrophobic aromatic residues such as W26, Y16, and F28, which have been experimentally shown to govern membrane insertion, oligomerization, and target binding. Similarly, the anti-inflammatory prediction selectively highlights residues implicated in protein–protein interactions and immunomodulatory signaling rather than broad membrane disruption. Notably, the antiviral prediction uniquely assigns elevated attention to G17, a residue known to participate in β-bulge formation and defensin dimerization, processes previously linked to viral neutralization mechanisms.

To further assess biological plausibility, we systematically mapped residues with attention to experimentally established molecular mechanisms reported in the literature (Table 2) [64]. This analysis demonstrates a strong correspondence between PepAnno’s learned representations and known structure–function relationships of HNP-1, including disulfide bond integrity, charge-mediated surface recognition, hydrophobic execution sites, and oligomerization-dependent activity. Residues receiving low attention predominantly localize to conserved β-sheet scaffolding regions, suggesting that the model appropriately distinguishes structural necessity from functional specificity.

**Table 2:** Residue-level mechanistic interpretation of PepAnno predictions for HNP-1.

Residue	Attention	Functional type	Established molecular mechanism	Supporting evidence from literature
**Identity Determinants and Folding/Assembly Core**				
**A1**	High	AMP, ACP, AAP, AIP, AVP	α-defensin identity determinant; N-terminal residue distinguishing HNP-1 from HNP-2 and HNP-3	HNP-1/2/3 differ only at the N-terminus; this position defines peptide identity and functional equivalence class
**A11**	High–Medium	AMP, ACP, AIP	Structural anchor within the β-sheet core; supports correct disulfide-bond geometry and oligomerization	α-defensin activity depends on intact β-sheet architecture stabilized by disulfide bonds
**C29, C30**	Medium	ACP	Disulfide bond formation; maintenance of tertiary structure required for biological activity	Reduction or alkylation of cysteines disrupts antimicrobial, antiviral, and immunomodulatory functions
**G17**	Medium–High	AVP	β-bulge formation and peptide dimerization; contributes to self-association	Gly17 is conserved across α-defensins and implicated in dimerization and oligomeric assembly
**Electrostatic Interaction Surface**				
**R14, R15**	High–Medium	AMP, AVP, AIP	Cationic surface mediating electrostatic interactions with anionic membranes and glycans	HNP-1 preferentially binds negatively charged microbial membranes and glycosylated surfaces
**R5**	Medium	CPP	Initial electrostatic contact with cell membranes; contributes to cellular uptake	Non-specific cationic interactions underlie defensin membrane association
**E13**	Medium	AMP, CPP	Salt-bridge formation with Arg5; enhances peptide stability in physiological environments	Arg5–Glu13 salt bridge increases resistance to proteolytic degradation without altering fold
**Hydrophobic Functional Execution Sites**				
**W26**	High	AAP	Hydrophobic aromatic hotspot; governs pathogen binding, dimerization, and functional versatility	Trp26 is critical for microbial binding, oligomerization, and multiple biological activities
**Y16**	High–Medium	AMP, ACP	Hydrophobic execution site involved in membrane interaction and cytotoxic activity	Tyr16 mutations demonstrate that side-chain hydrophobicity determines functional contribution
**Y3**	High	ACP, AIP, AAP	Aromatic surface residue contributing to protein–membrane and protein–protein interactions	Defensins destabilize pathogen proteins via exposure of hydrophobic patches
**F28**	Medium–High	ACP	Hydrophobic core residue contributing to membrane disruption and cytotoxicity	Phe28 mutagenesis supports hydrophobicity-dependent activity
**L25**	High	AIP	Hydrophobic determinant involved in immune modulation and target interaction	Leu25 mutations reduce functional activity, highlighting hydrophobic contribution
**I20**	Medium	AHP, AIP	Hydrophobic auxiliary residue influencing membrane-related interactions	Ile20 identified as part of the hydrophobic functional cluster
**I6, I10**	Medium	AVP, AIP	Supporting hydrophobic residues contributing to viral or protein interactions	Aliphatic side chains participate in target engagement
**Conformational Plasticity and Structural Adaptation Sites**				
**P7**	High	AAP, CPP, AHP	Conformational turn-inducing residue; introduces local structural flexibility	Proline residues modulate peptide geometry and functional adaptability
**G12, G23**	Medium–High	ACP, AAP, CPP	Flexible residues enabling surface adaptation and binding plasticity	Glycine residues facilitate β-bulges and conformational adjustments
**Q22**	Medium	AAP, CPP	Neutral polar residue contributing to surface presentation and target recognition	Polar side chains modulate binding without dominating electrostatics
**A27, A8**	Medium	AAP, CPP	Structural fine-tuning residues supporting local geometry	Small side chains accommodate compact β-sheet packing

Collectively, this case study illustrates that PepAnno not only recapitulates the known multifunctional repertoire of HNP-1 but also provides mechanistically interpretable insights at residue resolution. By aligning deep learning–derived attention with experimentally validated molecular mechanisms, PepAnno enables hypothesis-driven exploration of peptide function and offers a transparent framework for dissecting the functional complexity of bioactive peptides.

## Materials and methods

### Dataset construction

All datasets used in this study were collected from previously published studies to ensure fair and unbiased performance evaluation and comparison. In total, seven bioactivity-oriented BP datasets were curated, covering antimicrobial [65,66], anticancer [57], anti-inflammatory [58], antiviral [67], angiotensin-converting enzyme (ACE) inhibitory (anti-hypertensive) [68], anti-angiogenic [69], and cell-penetrating activities [70]. Detailed statistics and characteristics of each dataset are summarized in Table 3. Furthermore, to ensure the consistency of the feature space, we analyzed the sequence length distribution of the curated datasets. As visualized in Fig D in S1 Appendix, the length distributions of the training and independent test sets are highly consistent.

**Table 3:** Detailed information of datasets collected from publications.

Bioactivity	Train Dataset		Test Dataset	
	pos	neg	pos	neg
AMP	8387	8387	1536	1536
ACP	1176	1176	610	2760
AAP	107	107	28	28
AHP	856	856	214	363
AIP	1258	1887	420	629
AVP	2290	2311	417	395
CPP	844	838	90	98

For AMP dataset, we first merged the collected data and removed intra-dataset redundancies using CD-HIT [71] with a sequence identity threshold of 0.9. Because the independent test set reported by Xu et al. [66] was adopted for performance evaluation, we ensured strict separation by eliminating any AMP training sequences that exhibited ≥ 90% sequence identity to test set. Crucially, to prevent data leakage during the subsequent transfer learning process, we conducted explicit cross-task overlap checks. We utilized CD-HIT to remove any sequences from the AMP pre-training set that shared ≥ 90% identity with the independent test sets of the remaining six functional categories. This rigorous homology filtering yielded a final set of 8,387 positive AMP training sequences. Finally, to construct a balanced dataset, the negative samples were randomly down-sampled to 8,387 sequences, strictly matching the number of positive AMP samples used for model pre-training.

### PepAnno workflow

The PepAnno platform follows an end-to-end workflow encompassing data input, feature calculation, structural analysis, functional prediction, and result visualization (Fig 5). Users initiate the process by submitting peptide sequences and prediction parameters through the intuitive front-end interface. Upon submission, an automated quality control pipeline verifies compliance with FASTA format standards, with only validated data progressing to subsequent analysis.

**Fig 5:**
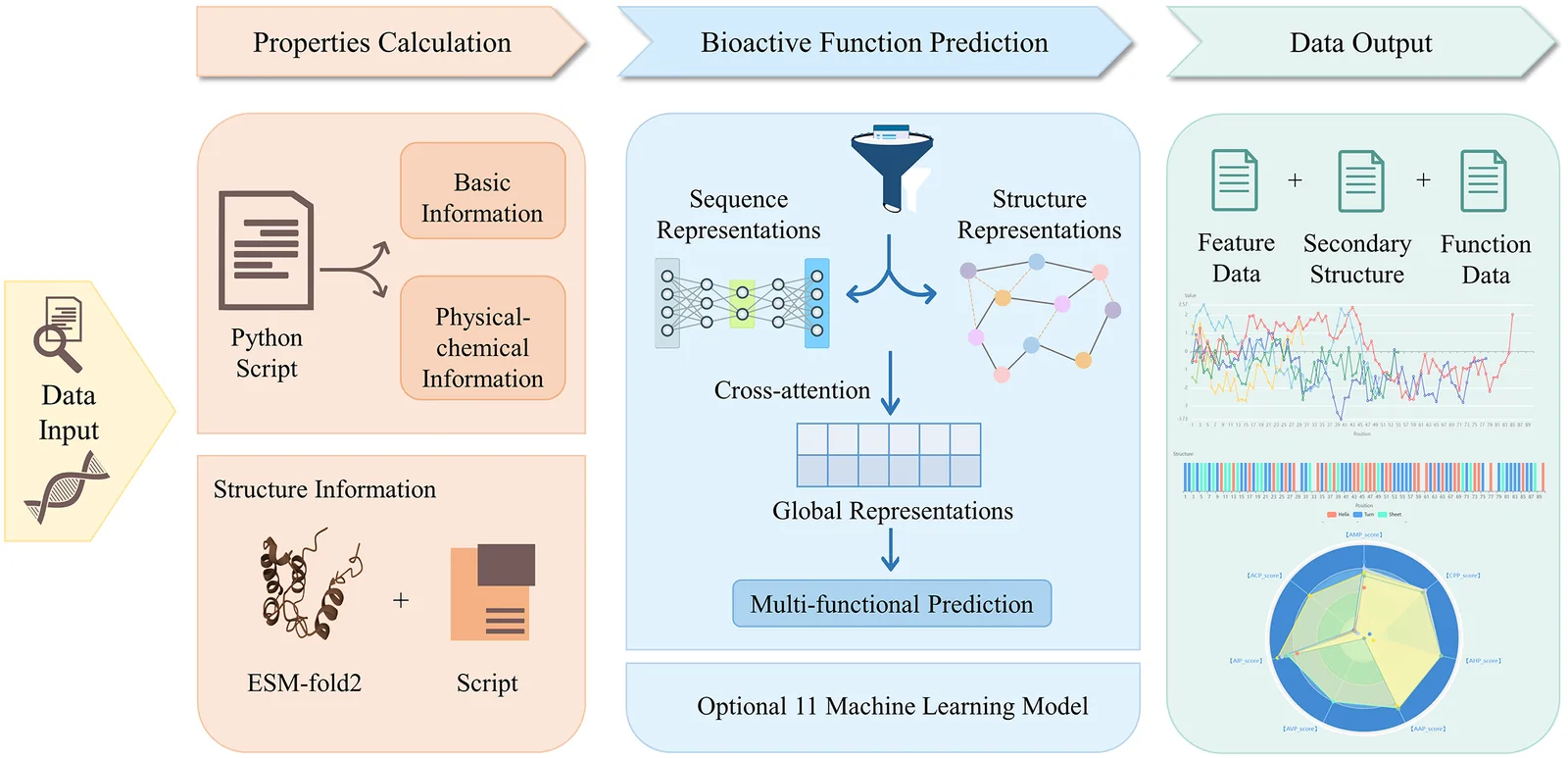
Backend workflow of the PepAnno platform. The process involves: (1) User data input (peptide sequences and parameters) followed by preprocessing. (2) Calculation of various peptide physicochemical features using toolkits. (3) Tertiary structure prediction of peptides. (4) Input of processed data into functional prediction model. (5) Final output of three main data files: comprehensive feature data, structural information, and integrated prediction results for all functions.

In the back-end pipeline, validated peptide sequences are first converted into SeqIO-compatible formats for standardized processing. PepAnno then performs systematic physicochemical feature calculation using established bioinformatics toolkits and internally developed scripts. This step generates a comprehensive feature profile, including basic sequence descriptors (e.g., peptide length and amino acid composition), core physicochemical properties (such as molecular weight, aromaticity, instability index, isoelectric point, extinction coefficients, GRAVY value, and flexibility), as well as 54 predefined amino acid–based scales derived from published studies. These multi-scale descriptors capture diverse chemical and biophysical characteristics of peptides and serve as essential inputs for downstream functional modeling and interpretation.

For structural analysis, PepAnno evaluates peptide secondary structure propensities by quantifying site-specific tendencies toward α-helix, β-turn, and β-sheet formation based on established amino acid preference models. The resulting secondary structure scores are used to generate position-resolved visualizations, facilitating intuitive inspection of local structural tendencies. In addition, PepAnno utilized ESMFold [72] to predict tertiary structure of peptides. The predicted three-dimensional models are integrated into the analysis pipeline and rendered through an interactive visualization module, enabling users to explore global folding patterns and spatial residue arrangements.

The analytical core of PepAnno is built upon a unified prediction framework that integrates multiple functional prediction modules corresponding to different bioactivity categories. Sequence-based representations, physicochemical features, and structural information are jointly utilized for feature extraction and classification within this framework. In addition to the proposed core model, the PepAnno platform also deploys a collection of 11 machine learning methods to support the prediction of seven bioactive peptide functions, providing complementary predictive perspectives (See Table K in S1 Appendix for details) [55,58–60,73–79]. The architecture and training strategy of the proposed core model are described in detail in the subsequent sections. For each functional category, the corresponding prediction module generates category-specific prediction scores, which are then systematically aggregated and organized within a unified analysis pipeline. The final results are presented through an integrated visualization interface, offering a comprehensive overview of predicted peptide functions across seven bioactivity categories and enabling efficient interpretation and comparative analysis of model outputs.

### Structure-aware multi-view deep learning framework

To achieve accurate identification and functional annotation of bioactive peptides across diverse categories, we propose a novel Structure-Aware Multi-view Geometric Deep Learning Framework (Fig 6). This framework synergistically integrates three core components: (1) a multi-view data representation module that constructs heterogeneous graphs from sequence and structural information; (2) a dual-stream neural architecture utilizing cross-modal attention for deep feature fusion; and (3) a strict hierarchical transfer learning strategy designed to ensure robust generalization on small-sample datasets.

**Fig 6:**
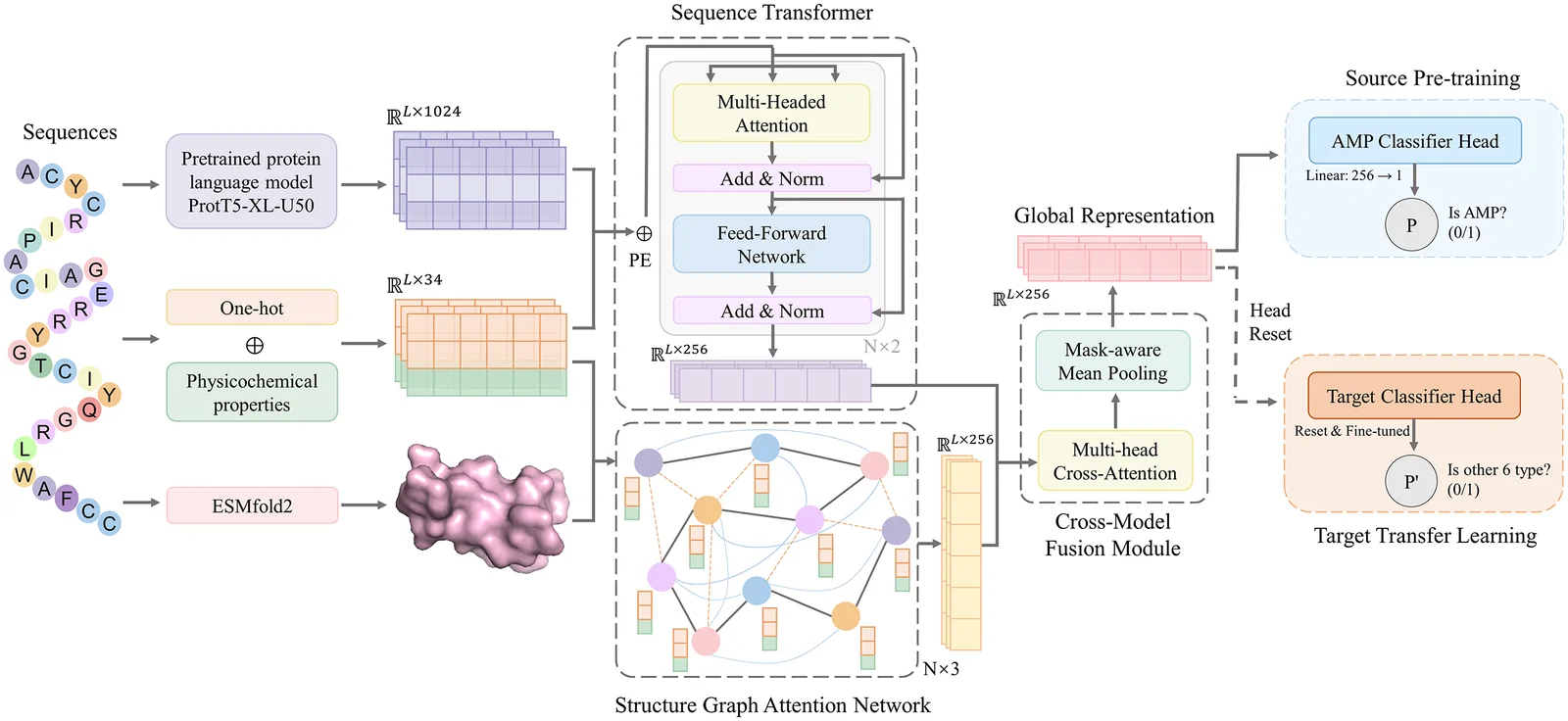
The overall illustration of PepAnno’s structure-aware multi-view geometric deep learning framework.

#### Data representation and heterogeneous graph construction.

To comprehensively capture the physicochemical and conformational characteristics of bioactive peptides, we represented each peptide sequence as both a sequential embedding and a geometric graph. For a peptide sequence of length *L*, we first predicted its three-dimensional (3D) structure using ESMFold2 and extracted the coordinates of *Cα* atoms. We constructed a heterogeneous biological graph G=(V,E) where nodes V represent amino acid residues. The node features ${\text{X}}_{\text{node}}\in R^{L\times \text{3}\text{4}}$ were composed of a 20-dimensional one-hot encoding of amino acid types concatenated with a 14-dimensional vector of physicochemical properties, including hydrophobicity, polarity, and van der Waals radius [58]. To model multi-scale interactions, the edge set E incorporated three distinct types of connections: primary edges connecting adjacent residues (i,i+1) to represent the peptide backbone; sequence window edges connecting residues within a local window (|i−j|≤2) to capture local sequential context; and structural kNN edges connecting the *k*-nearest neighbors (k=10) based on Euclidean distances between *Cα* atoms to encode long-range spatial dependencies critical for protein folding. Each edge was further featurized using Radial Basis Function (RBF) distance encodings, relative direction vectors, and positional encodings. Additionally, to leverage evolutionary information, we utilized the pre-trained ProtT5-XL-U50 model to extract residue-level embeddings, which were concatenated with ${\text{X}}_{\text{node}}$ to form a high-dimensional sequence representation ${\text{X}}_{\text{seq}}\in R^{L\times \text{1}\text{0}\text{5}\text{8}}$.

#### Multi-view geometric deep learning architecture.

The proposed model employs a dual-stream architecture to process the structural and sequential views in parallel. The structure stream utilizes a 3-layer GATv2 (Graph Attention Network v2) to process the graph G and node features ${\text{X}}_{\text{node}}$. By dynamically computing attention weights between neighboring residues, this stream updates node states to generate structural context tokens ${\text{H}}_{\text{struct}}\in R^{L\times \text{2}\text{5}\text{6}}$. Simultaneously, the sequence stream processes the high-dimensional representation ${\text{X}}_{\text{seq}}$ using a 2-layer Transformer Encoder. A padding mask is applied to handle variable-length sequences, allowing the mechanism to capture long-range semantic dependencies and output sequence tokens ${\text{H}}_{\text{seq}}\in R^{L\times \text{2}\text{5}\text{6}}$.

Instead of employing a simple late-concatenation strategy that prematurely collapses the spatial dimensions into an opaque black-box vector, we deliberately introduced a Cross-Modal Fusion module based on the cross-attention mechanism to integrate these two modalities. Specifically, the sequence tokens ${\text{H}}_{\text{seq}}$ serve as Queries, while the structural tokens ${\text{H}}_{\text{struct}}$ serve as Keys and Values. This design allows the semantic features to dynamically query relevant spatial contexts, effectively injecting geometric information into the sequence representation. Crucially, this cross-attention mechanism explicitly computes an L × L alignment matrix, thereby preserving the residue-level spatial resolution. Retaining this spatial dimension is fundamentally necessary for unlocking native 3D structural interpretability, enabling us to map predictive importance back to specific residues. The fused features are then aggregated via a mask-aware mean pooling layer into a global latent vector $\text{v}\in R^{\text{2}\text{5}\text{6}}$, which serves as the input for the final classification head.

#### Strict hierarchical transfer learning strategy.

Given the severe data imbalance between the abundant Antimicrobial Peptides (AMPs) and other functional categories with limited sample sizes (e.g., AAP, AIP), we implemented a strict hierarchical transfer learning strategy. In the first phase, designated as Source Domain Pre-training, the model backbone was trained on the balanced AMP dataset to learn generalized peptide feature representations. In the second phase, Target Domain Transfer, we utilized the pre-trained backbone weights to initialize models for specific target categories. Crucially, we employed a “Head Reset” strategy where the pre-trained linear classification head was discarded and re-initialized for each target task. This approach prevents negative transfer arising from the orthogonal decision boundaries of different functional classes. The model was then fine-tuned on the target datasets using the AdamW optimizer. Regarding the optimization objective, we employed advanced loss functions including Focal Loss and Poly Loss to address the imbalance between easy and hard samples. Specifically, Focal Loss was utilized to down-weight the contribution of well-classified examples and force the model to focus on “hard mining” of difficult samples near the decision boundary, while Poly Loss provided a flexible gradient adjustment framework based on Taylor expansion, enhancing generalization capabilities on small-scale datasets.

### Interpretability and visualization

To elucidate the biological basis of the model’s predictions, we analyzed the attention weights $\text{A}\in R^{L\times L}$ extracted from the cross-attention layer. We defined a Structure Importance Score for each residue by summing the attention weights received from all query tokens, representing the cumulative contribution of a specific structural region to the final prediction. To ensure robust visualization across different peptides, we applied percentile scaling to normalize these scores, clipping outliers to the 5th and 95th percentiles. These normalized scores were mapped to the B-factor field of the corresponding PDB files, allowing for the 3D visualization of high-attention regions. This method highlights functional motifs, such as active sites or hydrophobic cores, as regions of high importance, providing residue-level interpretability for the identified bioactive peptides.

### Resource curation

To establish a comprehensive and reliable knowledge base for the peptide research community, we conducted a systematic literature survey across WOS and PubMed, specifically targeting the seven bioactive functional categories addressed in this study. The search strategy employed combinations of bioactivity-specific terms with keywords such as “prediction”, “computational tool”. To facilitate efficient resource discovery and navigation within the platform, we implemented a data-driven taxonomy strategy based on keyword frequency analysis. For each curated resource, we manually extracted key descriptions summarizing its core algorithms and functionalities. These textual descriptions underwent tokenization and normalization processing to quantify the frequency of functional descriptors across the entire corpus. Based on this analysis, the ten most prevalent terms were selected as high-priority filter tags in the “Resources” module.

Complementing the tool repository, we also curated datasets which are categorized by functional type and made available for download through a dedicated “Data” interface [55,56,58–60,73–80], providing a centralized resource for researchers to benchmark new models or conduct meta-analyses.

### Server construction and implementation

The PepAnno platform is built on a robust architecture to ensure efficient data processing and a seamless user experience. Both the front-end and back-end are developed using the Django framework, with the user interface designed based on Bootstrap 5. Advanced visualization is supported through ECharts [81] and Mol* (MolStar) [82], enabling interactive and high-quality graphical representation. The platform is fully compatible with major web browsers, including Firefox, Google Chrome, and Microsoft Edge.

To ensure data security and user privacy, PepAnno adopts strict protection protocols. All communications between the client and server are encrypted via HTTPS. Submitted peptide sequences are used solely for the requested prediction tasks and are neither shared with third parties nor used for model retraining. Additionally, all uploaded data and generated results are stored temporarily and automatically deleted after 30 days, preventing long-term retention of sensitive information.

## Discussion and conclusion

In this study, we present PepAnno, a structure-aware and multi-functional peptide annotation platform designed to address both methodological and practical challenges in bioactive peptide analysis. Moving beyond conventional sequence-only predictors, PepAnno employs a dual-stream geometric deep learning architecture that synergizes pre-trained sequence semantics with 3D structural graphs via a cross-modal attention mechanism. To overcome the critical challenge of data scarcity and imbalance across different bioactivities, we implemented a strict hierarchical transfer learning strategy equipped with a “Head Reset” mechanism. Comprehensive benchmarking, rigorous ablation studies, and length-stratified evaluations demonstrate that PepAnno achieves highly robust and competitive performance, effectively avoiding negative transfer while maintaining strong out-of-distribution generalization. Crucially, rather than operating as an opaque black box, this architectural design unlocks native residue-level spatial interpretability, allowing researchers to visually pinpoint 3D functional motifs driving the bioactivity.

While our framework demonstrates robust generalization, it is important to delineate its applicability domain, particularly regarding sequence length. Architecturally, the dynamic nature of the graph and sequence attention mechanisms, combined with the mask-aware mean pooling layer, imposes no hard-coded limits on the input sequence length. However, the empirical predictive capability of the model is inherently bounded by the training data distribution. As illustrated in Fig D in S1 Appendix, the sequence lengths in our training and independent test sets are predominantly concentrated between 5 and 100 amino acids. Consequently, applying the model to significantly longer sequences (e.g., full-length proteins exceeding this spectrum) may lead to sub-optimal results. This performance degradation primarily occurs because the signals of localized functional motifs can be severely diluted by the vast non-functional background during the global pooling stage. Therefore, PepAnno is optimally suited for identifying sequences within the typical length spectrum of bioactive peptides.

Beyond predictive accuracy, PepAnno places strong emphasis on usability, accessibility, and workflow integration. The platform enables one-click, end-to-end peptide analysis without requiring programming expertise, substantially lowering the barrier to entry for experimental and translational researchers. By unifying physicochemical characterization, structural prediction, functional annotation, and resource integration within a single interface, PepAnno alleviates the fragmented workflows commonly encountered in peptide research and facilitates systematic exploration of peptide properties prior to downstream experimental validation.

In addition, PepAnno incorporates a curated repository that systematically aggregates peptide-related databases, computational tools, and web resources. This centralized design not only provides a comprehensive entry point for peptide research but also supports comparative analysis and hypothesis generation by enabling users to contextualize functional predictions within existing knowledge. As such, PepAnno serves not only as a predictive tool but also as an integrative knowledge platform for bioactive peptide research.

To further enhance the capabilities and utility of PepAnno in assessing peptides with therapeutic potential, we are committed to the continuous updating and improvement of our web platform in the following aspects:

(i) **Functional Expansion**: Firstly, we plan to integrate predictions for additional peptide functionalities within this web server. Secondly, we will incorporate target-related prediction capabilities in future updates, thereby providing resources for more detailed, mechanistic studies at a micro-level.
(ii) **Performance Optimization**: Beyond utilizing existing original models and datasets, we will persistently collect new data and explore novel methodologies to construct models with enhanced performance.

## Supporting information

S1 Appendix Supplementary Materials. This supporting document contains all supplementary tables and Figs cited in the main text. It includes the following sections: (a) Visualizations of PepAnno’s web interface. (b) Training dynamics and cross-validation stability. (c) Length distribution of datasets. (d) Overall performance of PepAnno. (e) Comparisons of multi-functional platforms. (f) Comparisons for 7 bioactive functions. (g) Detailed information about optional methods of PepAnno. (h) Evaluation Metrics.
(PDF)
