# Supplementary material for: PepAnno: A structure-aware deep learning framework for bioactive peptide prediction, structural visualization, and physicochemical profiling
Source: PLoS Comput Biol. 2026 Jun 2;22(6):e1014369. doi: 10.1371/journal.pcbi.1014369 (PMC13690271; doi:10.1371/journal.pcbi.1014369)
Supplement: Supplementary file 1 [file pcbi.1014369.s001.pdf]

PepAnno: A Structure-Aware Deep Learning Framework for Bioactive Peptide Prediction, Structural Visualization, and Physicochemical Profiling: Supplementary Materials

Enyan Liu[1], Yueming Hu[1], Liya Liu[1], Yifan Chen[1], Shilong Zhang[1], Sida Li[1], Haoyu Chao[1], Luyao Xie[1], Yi Shen[1], Liangwei Wu[1], Julio Raúl Fernández Massó[2] and Ming Chen[1,*]

[1] Department of Bioinformatics, College of Life Sciences, Zhejiang University, Hangzhou, 310058, China

[2] Pharmaceutical Department, Center for Genetic Engineering and Biotechnology, Havana, 10100, Cuba

[*] To whom correspondence should be addressed. Tel: +86 (0)571-88206612; Fax: +86 (0)571-88206612; Email: mchen@zju.edu.cn

Contents

1 Visualizations of PepAnno's web interface ... 3
2 Training dynamics and cross-validation stability ... 5
3 Length distribution of datasets ... 7
3 Overall performance of PepAnno ... 8
4 Comparisons of multi-functional platforms ... 9
5 Comparisons for 7 bioactive functions ... 10
6 Detailed information about optional methods of PepAnno ... 14
7 Evaluation Metrics ... 17
References: ... 18
List of legends of supporting figures and tables ... 21

1 Visualizations of PepAnno's web interface

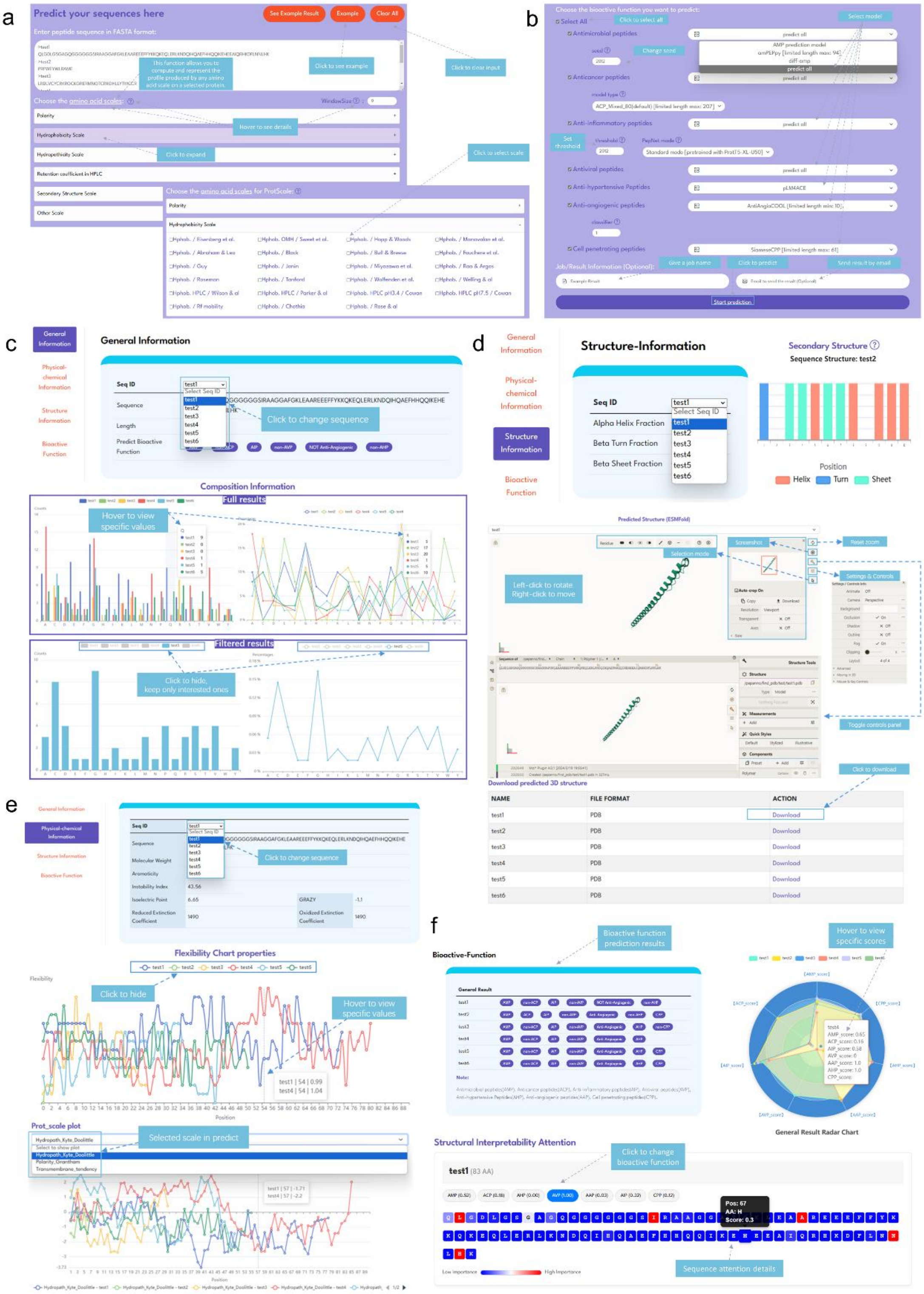

Figure A. Visualization PepAnno web interface. (a) The 'Predict' interface for peptide analysis. (b) The 'Other Tools' interface for optional model-based predictions. (c) General Information section,

including a summary table and amino acid composition visualizations. (d) Structural Information section, detailing secondary structure content and interactive 3D tertiary structure predictions. (e) Physicochemical Information section, displaying key properties with illustrative charts. (F) Bioactive Function section, summarizing predicted activities using radar charts and sequence-level attention profiles.

2 Training dynamics and cross-validation stability

As illustrated in figure B, the training process exhibits consistent convergence behavior across all functional categories. In panel(a), the training loss (left axis) decreases smoothly throughout the epochs, while the validation AUC (right axis) improves rapidly in the early stages and gradually stabilizes. Additionally, panel(b) present the validation AUC trajectories for all five folds. The shaded regions represent the standard deviation across folds. For most categories, the relatively narrow variance bands indicate strong robustness to data partitioning. Even in categories with larger variability, such as AAP and AIP, the model maintains consistent performance trends, suggesting reliable generalization.

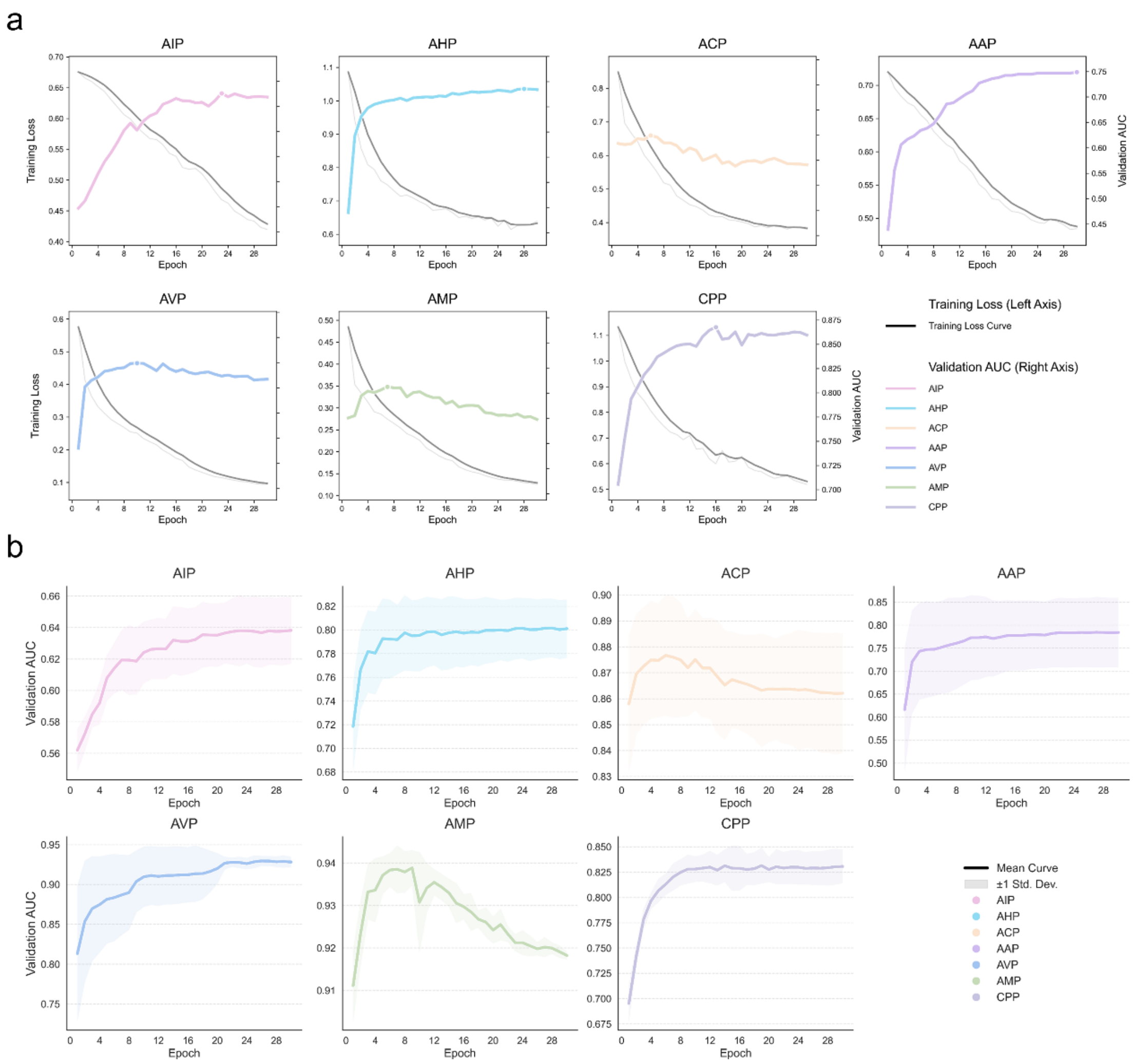

Figure B. (a) Training dynamics and convergence analysis. Each subplot represents the learning process for a specific functional category on its optimal fold. (b) Training dynamics across five folds. Each subplot illustrates the validation AUC progression over epochs for a specific functional category. Thin

lines represent individual folds, while the bold curve indicates the mean trajectory.

The performance across the 5 folds is summarized in table A and figure C. Overall, the model achieves strong and stable performance across all tasks. In particular, categories such as AVP and AMP exhibit both high predictive accuracy and low variance, indicating robust generalization. In contrast, relatively larger variance observed in AIP and AAP suggests increased task difficulty, likely due to limited sample size and higher intrinsic noise.

Table A. The overall performance of PepAnno under 5-fold cross-validation.

Bioactive type	ACC	AUC	MCC
AIP	0.63 ± 0.03	0.64 ± 0.02	0.19 ± 0.08
AHP	0.72 ± 0.02	0.80 ± 0.03	0.38 ± 0.07
ACP	0.82 ± 0.02	0.88 ± 0.02	0.64 ± 0.04
AAP	0.71 ± 0.09	0.79 ± 0.09	0.44 ± 0.17
AVP	0.86 ± 0.01	0.94 ± 0.01	0.73 ± 0.01
AMP	0.88 ± 0.01	0.94 ± 0.00	0.75 ± 0.01
CPP	0.76 ± 0.02	0.84 ± 0.02	0.52 ± 0.04

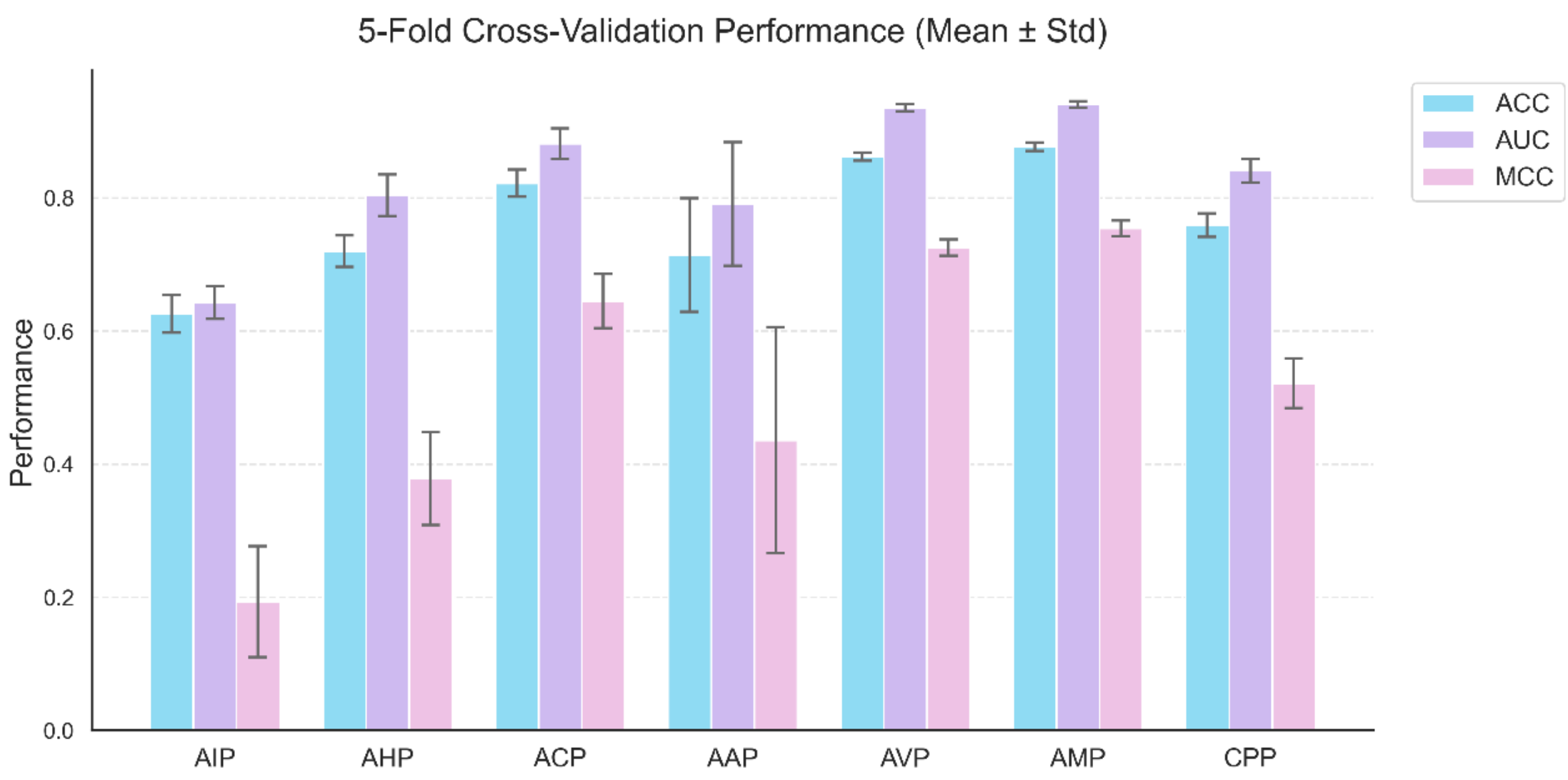

Figure C. 5-Fold cross-validation performance bar chart.

3 Length distribution of datasets

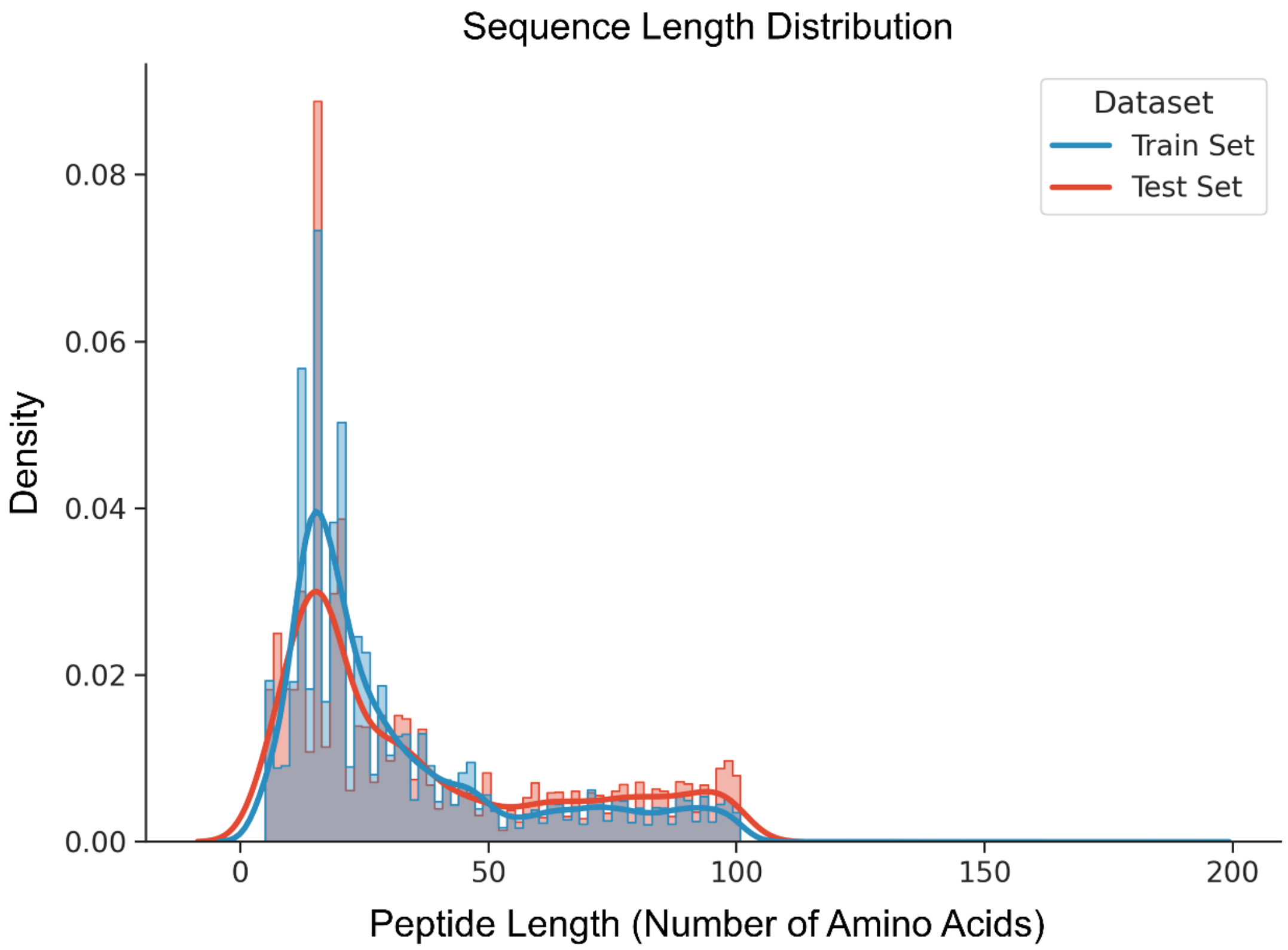

Figure D. Sequence length distribution of the curated datasets.

4 Overall performance of PepAnno

Table B. The overall performance of PepAnno on independent test data.

Bioactive Class	AUC	ACC	F1	Sn	Sp
AHP	0.79	0.73	0.62	0.61	0.80
AVP	0.94	0.88	0.88	0.89	0.87
AAP	0.66	0.61	0.54	0.46	0.75
CPP	0.91	0.85	0.84	0.86	0.84
AIP	0.66	0.67	0.55	0.50	0.78
ACP	0.85	0.83	0.60	0.70	0.85
AMP	0.77	0.71	0.64	0.52	0.89

5 Comparisons of multi-functional platforms

Table C. Comparison of multi-functional bioactive peptide prediction platforms on independent test data.

Bioactive type	Tool	ACC	SN	SP	PRE	F1	MCC	AUC
ACP	PepAnno	**0.83**	**0.70**	0.85	**0.52**	**0.60**	**0.50**	**0.85**
	autopeptideml	0.78	0.06	**0.94**	0.18	0.09	0.00	0.54
	UniDL4BioPep(main)	0.60	0.52	0.62	0.23	0.32	0.11	0.58
	UniDL4BioPep(alt)	0.79	0.54	0.85	0.44	0.48	0.36	0.80
	iAMPCN	0.25	**0.70**	0.15	0.15	0.25	(0.16)	-
AHP	PepAnno	**0.73**	0.61	**0.80**	**0.64**	**0.62**	**0.41**	**0.79**
	autopeptideml	0.66	0.41	0.82	0.56	0.47	0.24	0.67
	UniDL4BioPep	0.61	**0.65**	0.58	0.50	0.56	0.22	0.63
AMP	PepAnno	0.71	0.52	0.89	0.83	0.64	0.45	**0.77**
	autopeptideml	0.52	0.48	0.56	0.52	0.50	0.04	0.52
	UniDL4BioPep	0.67	0.40	0.94	0.86	0.54	0.40	0.72
	iAMPCN	**0.81**	**0.68**	**0.94**	**0.92**	**0.79**	**0.65**	-
AVP	PepAnno	**0.88**	**0.89**	**0.87**	**0.88**	**0.88**	**0.76**	**0.94**
	autopeptideml	0.61	0.65	0.56	0.61	0.63	0.22	0.62
	UniDL4BioPep	0.85	0.85	0.84	0.85	0.85	0.69	0.91
	iAMPCN	0.66	0.87	0.44	0.62	0.73	0.35	-

6 Comparisons for 7 bioactive functions

Table D. Performance comparison for antimicrobial peptide (AMP) prediction on independent test data.

Tool	ACC	MCC	SN	SP
amPEPpy	0.68	0.43	0.40	0.96
AMPfun	0.67	0.41	0.41	0.94
AMPEP	0.66	0.43	0.33	**0.99**
ADAM-HMM	0.68	0.39	0.52	0.85
Ampir	0.56	0.16	0.27	0.86
AMPScannerV2	0.57	0.14	0.52	0.61
AmpGram	0.56	0.13	0.45	0.68
Deep-AMPEP30	0.53	0.18	0.07	1.00
CAMP-ANN	0.58	0.18	0.39	0.78
sAMPpred-GAT	0.71 ± 0.01	0.46 ± 0.01	**0.53 ± 0.04**	0.90 ± 0.02
PepAnno	**0.71**	**0.45**	0.52	0.89

Table E. Performance comparison for anticaner peptide (ACP) prediction on independent test data.

Tool	MCC	SN	SP	PRE	ACC	AUC	F1
PepAnno	0.50	0.70	0.85	0.52	**0.83**	**0.85**	**0.60**
iAMPCN	(0.21)	0.63	0.16	0.14	0.24	0.68	0.23
mACPpred	**0.49**	0.75	0.82	0.48	0.81	0.84	0.59
ACP-MHCNN (740)	0.13	0.36	0.79	0.27	0.71	-	0.31
ACP-MHCNN (500/164)	(0.10)	0.17	0.72	0.12	0.62	-	0.14
ACPred-BMF (Main)	0.20	0.67	0.58	0.26	0.60	0.33	0.38
ACPred-BMF (Alternate)	0.25	0.44	0.83	0.36	0.76	0.26	0.40
ACPred	0.28	0.51	0.80	0.37	0.75	0.74	0.43
AMPfun	0.43	**0.77**	0.76	0.41	0.76	0.80	0.54
iAMP-RAAC	0.10	0.07	0.98	0.41	0.81	-	0.11
AntiCP 2.0 (Main)	0.18	0.43	0.77	0.30	0.71	0.60	0.35
AntiCP 2.0 (Alternate)	0.47	0.61	0.89	0.54	0.84	0.84	0.57
CancerGram	0.11	0.07	0.98	0.44	0.82	0.67	0.11
iDACP (Sp of 90%)	0.26	0.20	0.96	**0.55**	**0.83**	**0.85**	0.30
iDACP (Sp of 80%)	0.33	0.36	0.93	0.52	0.82	**0.85**	0.42
iDACP (Sp of 70%)	0.38	0.48	0.89	0.50	0.82	**0.85**	0.49

TriNet	0.23	0.43	0.82	0.34	0.75	0.72	0.38
PreTP-2L	0.07	0.02	**0.99**	0.45	0.82	**-**	0.04

Table F. Performance comparison for anti-inflammatory peptide (AIP) prediction on independent test data.

Tool	ACC	SN	PRE	F1	MCC
PepAnno	0.67	0.50	0.60	0.55	0.29
AIPStack	0.74	0.61	0.70	0.65	0.45
PPTPP (Class feature)	0.71	0.52	0.68	0.59	0.38
PPTPP (Probability feature)	0.73	0.56	0.70	0.62	0.42
PPTPP (Fusion feature)	0.63	0.10	**0.78**	0.17	0.18
AIP_MDL	0.76	0.73	0.77	0.71	0.50
TriNet	0.67	0.45	0.62	0.52	0.29
PepNet (Standard)	**0.82**	**0.94**	0.71	**0.81**	**0.67**
PepNet (Fast)	0.76	0.78	0.68	0.72	0.52

Table G. Performance comparison for antiviral peptide (AVP) prediction on independent test data.

Tool	ACC	SN	SP	MCC	PRE	AUC
PepAnno	0.88	0.89	0.87	0.76	0.88	0.94
ENNAVIA	0.51	0.52	0.51	0.03	0.56	0.49
PreTP-Stack	0.52	0.39	0.66	0.05	0.55	0.53
DeepAVP	0.52	0.59	**0.99**	0.15	0.54	0.53
iAMP-CA2L	0.53	0.06	0.47	0.06	0.89	0.53
Meta-iAVP	0.58	0.59	0.57	0.15	0.59	0.58
AVPIden	0.60	0.74	0.47	0.21	0.57	0.69
iACVP	0.66	0.47	0.86	0.35	0.77	0.76
Deep-AVPiden	**0.90**	**0.90**	0.92	**0.80**	**0.90**	**0.96**

Table H. Performance comparison for antihypertensive peptide (AHP) prediction on independent test data.

Tool	PRE	SP	SN	ACC	MCC	F1	AUC
PepAnno	0.64	0.80	0.61	0.73	0.41	0.62	0.79
AHPCG	0.64	0.77	0.70	0.75	0.46	0.67	0.83
AHPLSTM	0.63	0.77	0.67	0.73	0.44	0.65	0.79
AHTpin	0.67	0.84	0.55	0.73	0.41	0.60	0.81
AHPred-DE	0.67	0.81	0.65	0.75	0.46	0.66	0.80
pLM4ACE	0.69	0.85	0.58	0.75	0.44	0.63	0.80
AI4ACEIP	**0.74**	**0.85**	**0.71**	**0.81**	**0.59**	**0.75**	**0.90**

Table I. Performance comparison for anti-angiogenic peptide (AAP) prediction on independent test data.

Tool	AUC	ACC	SN	SP	PRE	F1	MCC
PepAnno	**0.66**	**0.61**	0.46	**0.75**	**0.65**	0.54	**0.22**
AntAngioCOOL	0.60	0.50	**1.00**	0.00	0.50	**0.67**	0.00

Table J. Performance comparison for cell-penetrating peptide (CPP) prediction on independent test data.

Tool	MCC	ACC	PRE	SN	F1	AUC
GraphCPP	0.58	0.80	0.78	0.73	0.75	0.85
MLCPP2	0.30	0.67	0.79	0.26	0.39	0.69
CellPPD (SVM + motif)	0.22	0.64	0.69	0.21	0.32	-
CellPPD-mod (SVM)	0.20	0.63	0.57	0.40	0.47	-
C2Pred	0.05	0.57	0.46	0.26	0.33	0.65
CellPPD (SVM)	0.00	0.59	0.00	0.00	0.00	-
PepAnno	**0.69**	**0.85**	**0.80**	**0.86**	**0.84**	**0.91**

7 Detailed information about optional methods of PepAnno

Table K. Overview of detailed information about optional computational methods of PepAnno platform.

Functions	Methods	Computational method	Data source	Positive data	Negative data
Antimicrobial peptides	amPEPpy[1]	Random forest	APD3 [2], CAMPR3 [3] and LAMP databases [4]	3268 AMP sequences	166791 non-AMP sequences from UniProt
	Diff-amp[5]	Pre-training and transfer learning	Dataset1, sourced from the CAMP server [6], dataset2 [7].	Dataset1 includes 8225 AMP sequences primarily utilized for AMP generation and iterative optimization training. Dataset2 includes 4134 AMP and 4134 non-AMP samples.	
Anticancer peptides	acp-ope[8]	LightGBM, Bi-LSTM and CNN	benchmark paper [9]	859 ACPs retrieved from CancerPPD database [10]	859 non-ACPs from the antimicrobial peptides.
	con_ACP[11]	Contrastive learning	six benchmark datasets organized by He et al. [12-16]	-	-
Anti-inflammatory peptides	PREDAIP[17]	emRMR-SFS and 7 classifiers	datasets from the recently published articles and	2139 positive	3327 negative samples

			IEDB database [18].	samples	
	PepNet[19]	Pre-trained protein model and residual Transformer blocks	from research [20, 21]	peptides from the Immune Epitope Database (IEDB) [18]	Those that fail to induce the negative samples.
Antiviral peptides	ACVPred[22]	Transformer with transfer learning and data augmentation	collected a total of 284 experimentally validated ACVPs from the ACovPepDB database [23] and two studies [24, 25].	1248 AVPs, 154 ACVPs	6296 non-AVPs
	FFMAVP[26]	Feature fusion and multitask learning.	based on Pang et al.'s dataset [27].	2662 AVPs and 10 095 non-AVPs that were from databases such as AVPdb [28], HIPdb [29], UniProt [30] and classified AVPs into specific classes for virus families and species, respectively.	
antihypertensive peptide	pLM4ACE[31]	Logistic regression and ESM-2 among the 12 embeddings and 5 ML models	From BIOPEP-UWM [32], AHTPDB [33], and 267 published papers.	394 peptides were in the high activity group and 626 peptides were in the low & non-activity group	
anti-angiogenic peptides	AntAngioCOOL[34]	3 classifiers from 227 tested machine learning models	the gold standard dataset that has been published [35].	135 anti-angiogenic peptides	135 non anti-angiogenic peptides

cell-penetrating peptides	SiameseCPP[36]	Pretrained model and a Siamese neural network	Based on the publicly available dataset CPP924 [37].	462 experimentally validated CPPs	462 non-CPPs as negative samples

8 Evaluation Metrics

To evaluate the classification performance of the proposed model, we employed a set of standard metrics derived from the confusion matrix. Let TP (True Positive), TN (True Negative), FP (False Positive), and FN (False Negative) denote the number of correctly classified positive samples, correctly classified negative samples, incorrectly classified positive samples, and incorrectly classified negative samples, respectively. Based on these terms, the metrics including Accuracy (ACC), Sensitivity (Sn), Specificity (Sp), Precision (PRE), and F1-score are defined as follows:

$$\mathrm{ACC} = \frac{\mathrm{TP} + \mathrm{TN}}{\mathrm{TP} + \mathrm{TN} + \mathrm{FP} + \mathrm{FN}}$$

$$\mathrm{Sn} = \frac{\mathrm{TP}}{\mathrm{TP} + \mathrm{FN}}$$

$$\mathrm{Sp} = \frac{\mathrm{TN}}{\mathrm{TN} + \mathrm{FP}}$$

$$\mathrm{PRE} = \frac{\mathrm{TP}}{\mathrm{TP} + \mathrm{FP}}$$

$$\mathrm{F1} = \frac{2 \times \mathrm{PRE} \times \mathrm{Sn}}{\mathrm{PRE} + \mathrm{Sn}}$$

Furthermore, we specifically utilized the Matthews Correlation Coefficient (MCC) as a primary metric due to its robustness in binary classification tasks, as it provides a balanced measure even if the classes are of very different sizes:

$$\mathrm{MCC} = \frac{\mathrm{TP} \times \mathrm{TN} - \mathrm{FP} \times \mathrm{FN}}{\sqrt{(\mathrm{TP} + \mathrm{FP})(\mathrm{TP} + \mathrm{FN})(\mathrm{TN} + \mathrm{FP})(\mathrm{TN} + \mathrm{FN})}}$$

In addition to threshold-dependent metrics, we also calculated the Area Under the Receiver Operating Characteristic Curve (AUC). The AUC score measures the model's discriminative ability across all possible classification thresholds, providing a threshold-independent evaluation of performance.

References:

1. Lawrence TJ, Carper DL, Spangler MK, Carrell AA, Rush TA, Minter SJ et al. amPEPpy 1.0: a portable and accurate antimicrobial peptide prediction tool, Bioinformatics 2021;37:2058-2060.

2. Wang G, Li X, Wang Z. APD3: the antimicrobial peptide database as a tool for research and education, Nucleic Acids Research 2016;44:D1087-D1093.

3. Waghu FH, Barai RS, Gurung P, Idicula-Thomas S. CAMPR3: a database on sequences, structures and signatures of antimicrobial peptides, Nucleic Acids Research 2016;44:D1094-D1097.

4. Zhao X, Wu H, Lu H, Li G, Huang Q. LAMP: A Database Linking Antimicrobial Peptides, Plos One 2013;8.

5. Wang R, Wang T, Zhuo L, Wei J, Fu X, Zou Q et al. Diff-AMP: tailored designed antimicrobial peptide framework with all-in-one generation, identification, prediction and optimization, Brief Bioinform 2024;25.

6. Wang Q, Yuan Y. Learning to resize image, Neurocomputing 2014;131:357-367.

7. Cao Q, Ge C, Wang X, Harvey PJ, Zhang Z, Ma Y et al. Designing antimicrobial peptides using deep learning and molecular dynamic simulations, Briefings in Bioinformatics 2023;24.

8. Yuan Q, Chen K, Yu Y, Le NQK, Chua MCH. Prediction of anticancer peptides based on an ensemble model of deep learning and machine learning using ordinal positional encoding, Brief Bioinform 2023;24.

9. Lv Z, Cui F, Zou Q, Zhang L, Xu L. Anticancer peptides prediction with deep representation learning features, Briefings in Bioinformatics 2021;22.

10. Tyagi A, Tuknait A, Anand P, Gupta S, Sharma M, Mathur D et al. CancerPPD: a database of anticancer peptides and proteins, Nucleic Acids Research 2015;43:D837-D843.

11. Lee B, Shin D. Contrastive learning for enhancing feature extraction in anticancer peptides, Brief Bioinform 2024;25.

12. Yi H-C, You Z-H, Zhou X, Cheng L, Li X, Jiang T-H et al. ACP-DL: A Deep Learning Long Short-Term Memory Model to Predict Anticancer Peptides Using High-Efficiency Feature Representation, Molecular Therapy Nucleic Acids 2019;17:1-9.

13. Wei L, Zhou C, Chen H, Song J, Su R. ACPred-FL: a sequence-based predictor using effective feature representation to improve the prediction of anti-cancer peptides, Bioinformatics 2018;34:4007-4016.

14. Agrawal P, Bhagat D, Mahalwal M, Sharma N, Raghava GPS. AntiCP 2.0: an updated model for predicting anticancer peptides, Briefings in Bioinformatics 2021;22.

15. He W, Wang Y, Cui L, Su R, Wei L. Learning embedding features based on multisense-scaled

attention architecture to improve the predictive performance of anticancer peptides, Bioinformatics 2021;37:4684-4693.

16. Manavalan B, Basith S, Shin TH, Choi S, Kim MO, Lee G. MLACP: machine-learning-based prediction of anticancer peptides, Oncotarget 2017;8:77121-77136.

17. Lin D, Yu JL, Zhang J, He H, Guo XY, Shi SP. PREDAIP: Computational Prediction and Analysis for Anti-inflammatory Peptide<i> via</i> a Hybrid Feature Selection Technique, Current Bioinformatics 2021;16:1048-1059.

18. Vita R, Mahajan S, Overton JA, Dhanda SK, Martini S, Cantrell JR et al. The Immune Epitope Database (IEDB): 2018 update, Nucleic Acids Research 2019;47:D339-D343.

19. Han J, Kong T, Liu J. PepNet: an interpretable neural network for anti-inflammatory and antimicrobial peptides prediction using a pre-trained protein language model, Commun Biol 2024;7:1198.

20. Guan J, Yao L, Chung C-R, Xie P, Zhang Y, Deng J et al. Predicting Anti-inflammatory Peptides by Ensemble Machine Learning and Deep Learning, Journal of Chemical Information and Modeling 2023;63:7886-7898.

21. Gupta S, Sharma AK, Shastri V, Madhu MK, Sharma VK. Prediction of anti-inflammatory proteins/peptides: an insilico approach, J Transl Med 2017;15:7.

22. Xu Y, Liu TY, Yang Y, Kang JJ, Ren LP, Ding H et al. ACVPred: Enhanced prediction of anti-coronavirus peptides by transfer learning combined with data augmentation, Future Generation Computer Systems-the International Journal of Escience 2024;160:305-315.

23. Zhang Q, Chen X, Li B, Lu C, Yang S, Long J et al. A database of anti-coronavirus peptides, Scientific Data 2022;9.

24. Kurata H, Tsukiyama S, Manavalan B. iACVP: markedly enhanced identification of anti-coronavirus peptides using a dataset-specific word2vec model, Briefings in Bioinformatics 2022;23.

25. Pang Y, Wang Z, Jhong J-H, Lee T-Y. Identifying anti-coronavirus peptides by incorporating different negative datasets and imbalanced learning strategies, Briefings in Bioinformatics 2021;22:1085-1095.

26. Cao R, Hu W, Wei P, Ding Y, Bin Y, Zheng C. FFMAVP: a new classifier based on feature fusion and multitask learning for identifying antiviral peptides and their subclasses, Brief Bioinform 2023;24.

27. Pang Y, Yao L, Jhong J-H, Wang Z, Lee T-Y. AVPIden: a new scheme for identification and functional prediction of antiviral peptides based on machine learning approaches, Briefings in Bioinformatics 2021;22.

28. Qureshi A, Thakur N, Tandon H, Kumar M. AVPdb: a database of experimentally validated antiviral peptides targeting medically important viruses, Nucleic Acids Research 2014;42:D1147-D1153.

29. Qureshi A, Thakur N, Kumar M. HIPdb: A Database of Experimentally Validated HIV Inhibiting Peptides, Plos One 2013;8.

30. Bateman A, Martin M-J, Orchard S, Magrane M, Alpi E, Bely B et al. UniProt: a worldwide hub of protein knowledge, Nucleic Acids Research 2019;47:D506-D515.

31. Du Z, Ding X, Hsu W, Munir A, Xu Y, Li Y. pLM4ACE: A protein language model based predictor for antihypertensive peptide screening, Food Chem 2024;431:137162.

32. Minkiewicz P, Iwaniak A, Darewicz M. BIOPEP-UWM Database of Bioactive Peptides: Current Opportunities, International Journal of Molecular Sciences 2019;20.

33. Kumar R, Chaudhary K, Sharma M, Nagpal G, Chauhan JS, Singh S et al. AHTPDB: a comprehensive platform for analysis and presentation of antihypertensive peptides, Nucleic Acids Research 2015;43:D956-D962.

34. Zahiri J, Khorsand B, Yousefi AA, Kargar M, Shirali Hossein Zade R, Mahdevar G. AntAngioCOOL: computational detection of anti-angiogenic peptides, J Transl Med 2019;17:71.

35. Ramaprasad ASE, Singh S, Gajendra RPS, Venkatesan S. AntiAngioPred: A Server for Prediction of Anti-Angiogenic Peptides, Plos One 2015;10.

36. Zhang X, Wei L, Ye X, Zhang K, Teng S, Li Z et al. SiameseCPP: a sequence-based Siamese network to predict cell-penetrating peptides by contrastive learning, Brief Bioinform 2023;24.

37. Wei L, Tang J, Zou Q. SkipCPP-Pred: an improved and promising sequence-based predictor for predicting cell-penetrating peptides, Bmc Genomics 2017;18.

List of legends of supporting figures and tables

- **Figure A.** Visualization PepAnno web interface.
- **Figure B.** Training dynamics and cross-validation stability.
- **Figure C.** 5-Fold cross-validation performance bar chart.
- **Figure D.** Sequence length distribution of the curated datasets.
- **Table A.** The overall performance of PepAnno under 5-fold cross-validation.
- **Table B.** The overall performance of PepAnno on independent test data.
- **Table C.** Comparison of multi-functional bioactive peptide prediction platforms on independent test data.
- **Table D.** Performance comparison for antimicrobial peptide (AMP) prediction on independent test data.
- **Table E.** Performance comparison for anticaner peptide (ACP) prediction on independent test data.
- **Table F.** Performance comparison for anti-inflammatory peptide (AIP) prediction on independent test data.
- **Table G.** Performance comparison for antiviral peptide (AVP) prediction on independent test data.
- **Table H.** Performance comparison for antihypertensive peptide (AHP) prediction on independent test data.
- **Table I.** Performance comparison for anti-angiogenic peptide (AAP) prediction on independent test data.
- **Table J.** Performance comparison for cell-penetrating peptide (CPP) prediction on independent test data.
- **Table K.** Overview of detailed information about optional computational methods of PepAnno platform.
